# Mobile Workflow in Computed Tomography of the Chest

**DOI:** 10.1007/s10916-018-1131-2

**Published:** 2018-12-10

**Authors:** Matthias Wetzl, Melanie Weller, Rafael Heiss, Eleni Schrüfer, Wolfgang Wuest, Carsten Thierfelder, Daniel Lerch, Alexander Cavallaro, Patrick Amarteifio, Michael Uder, Matthias Stefan May

**Affiliations:** 1 Department of Radiology, University Hospital Erlangen, Maximiliansplatz 3, 91054 Erlangen, Germany; 2 Siemens Healthcare GmbH, Siemensstr. 3, 91301 Forchheim, Germany; 3 Imaging Science Institute, University Hospital Erlangen, Ulmenweg 18, 91054 Erlangen, Germany; 4 Siemens Healthcare GmbH, Henkestr. 127, 91052 Erlangen, Germany

**Keywords:** X-ray computed tomography, Mobile workflow, Thorax, Tablets, Heatmaps

## Abstract

A CT system with a tablet as mobile user interface and a wireless remote control for positioning and radiation release has recently been presented. Our aim was to evaluate the effects of a mobile CT examination workflow on the radiographers’ performance compared to conventional examinations. A prototype of a radiation protection cabin was installed besides the gantry of a CT system. The CT system was equipped with a simplified user interface on a portable tablet and a mobile remote control. 98 patients with an indication for CT of the chest were randomly assigned to examination using the mobile devices (study group, *n* = 47) or using the conventional stationary workflow on the console (reference group, *n* = 51). Three ceiling mounted fisheye cameras were used for motion tracking of the radiographers, two in the examination room and one in the control room. Relative density of detection heat-maps and area counts were assessed using a dedicated software tool to quantify radiographers’ movements. Duration of each task of the examination was manually recorded using a stopwatch. In the reference group 25% of the area counts were located inside of the examination room, while it was 48% in the study group. The time spent in the same room with the patient increased from 3:06 min (29%) to 6:01 min (57%) using the mobile workflow (*p* < 0.05), thereof 0:59 min (9%) were spent in moderate separation with maintained voice and visual contact in the radiation protection cabin. Heat-maps showed an increase of the radiographer’s working area, indicating a higher freedom of movement. Total duration of the examination was slightly less in the study group without statistical significance (median time: study 10:36, reference 10:50 min; *p* = 0.29). A mobile CT examination transfers the radiographers’ interaction with the scanner from the control room into the examination room. There, radiographers’ freedom of movement is higher, without any tradeoffs regarding the examination duration.

## Introduction

Digitalization and the introduction of radiological information systems (RIS) and picture archiving and communication systems (PACS) allowed for digital image interpretation and established a linear workflow operating a computed tomography (CT) [1]. Advancements in hardware (tube, detector, or gantry) and software components (reconstruction algorithms, dose saving algorithms) evolved to faster acquisitions with higher resolutions at reduced radiation doses [2]. However, the CT acquisition sequence almost stayed the same until today: registration – positioning – planning – examination – release – reconstruction – archiving. Patient contact of the medical staff is limited to positioning and release of the patient in the examination room during this sequence. The majority of settings and adjustments are controlled from an operating console in a separate control room, where communication is limited to microphones and speakers. This separation between patients and medical staff strongly interferes with efforts to enforce compliance, especially in uncooperative patients. Injured patients, critically ill patients, demented patients or pediatric patients are especially at risk and may require assistance and surveillance during the entire examination, also during radiation [3]. Additional staff in the examination room, wrapped up with lead aprons, is still the only solution to overcome these situations. However, additional human resources are often limited, especially during nightshifts, weekends and holidays, in rural areas and in underdeveloped countries.

Mobile devices are already established in the consumer market and are also increasingly used for home medicine applications [4]. Since they are continuously improving in computing power and battery capacity, many implementations have recently been evaluated in radiologic departments, e.g. for patient briefing [5], diagnostic procedures [6], clinical knowledge assistance [7], case database management [8] or augmented reality for interventional procedures [9]. Recently a complete user interface application for mobile tablet devices has been presented for CT systems. A redesign of the examination workflow, bringing the radiographer closer to the patient, seems to be possible by integrating these mobile devices in the daily clinical routine. The aim of this study was to evaluate if the time spent together with the patient, which we consider as surrogate for patient contact and interaction, can be increased for radiographers using a mobile workflow to operate a CT of the chest in comparison to the conventional stationary console workflow. Patient proximity and patient experience are hardly measurable parameters. Therefore, camera surveillance and chronographic measurements were chosen for assessment of the surrogate. A prototype of x-ray protection cabin was additionally designed for this study to avoid the time consuming and inconvenient process of dressing and undressing lead aprons.

## Materials and methods

### Mobile workflow

**Fig. 1:**
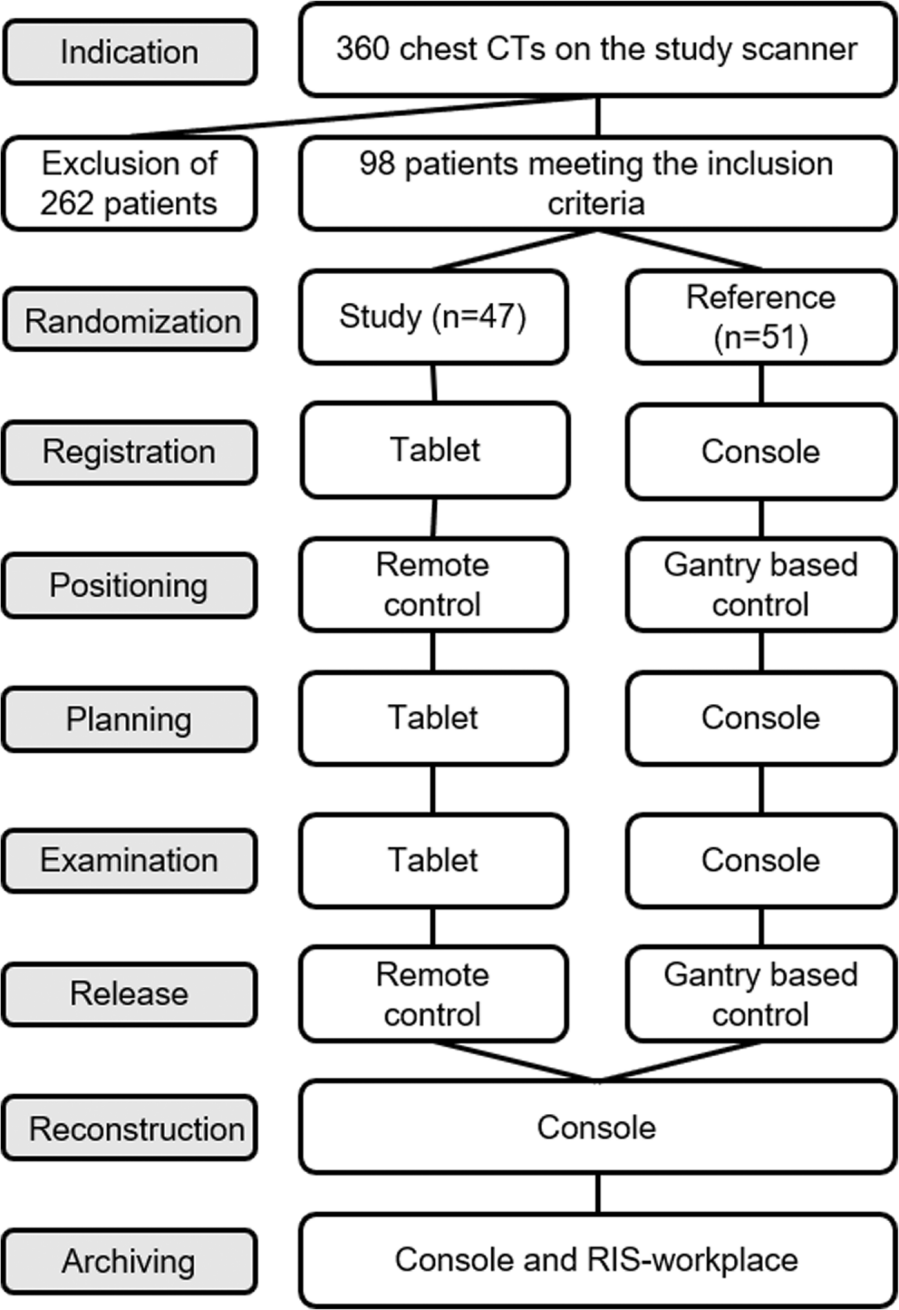
Flowchart of the examination workflow for the study group and the reference group

**Fig. 2:**
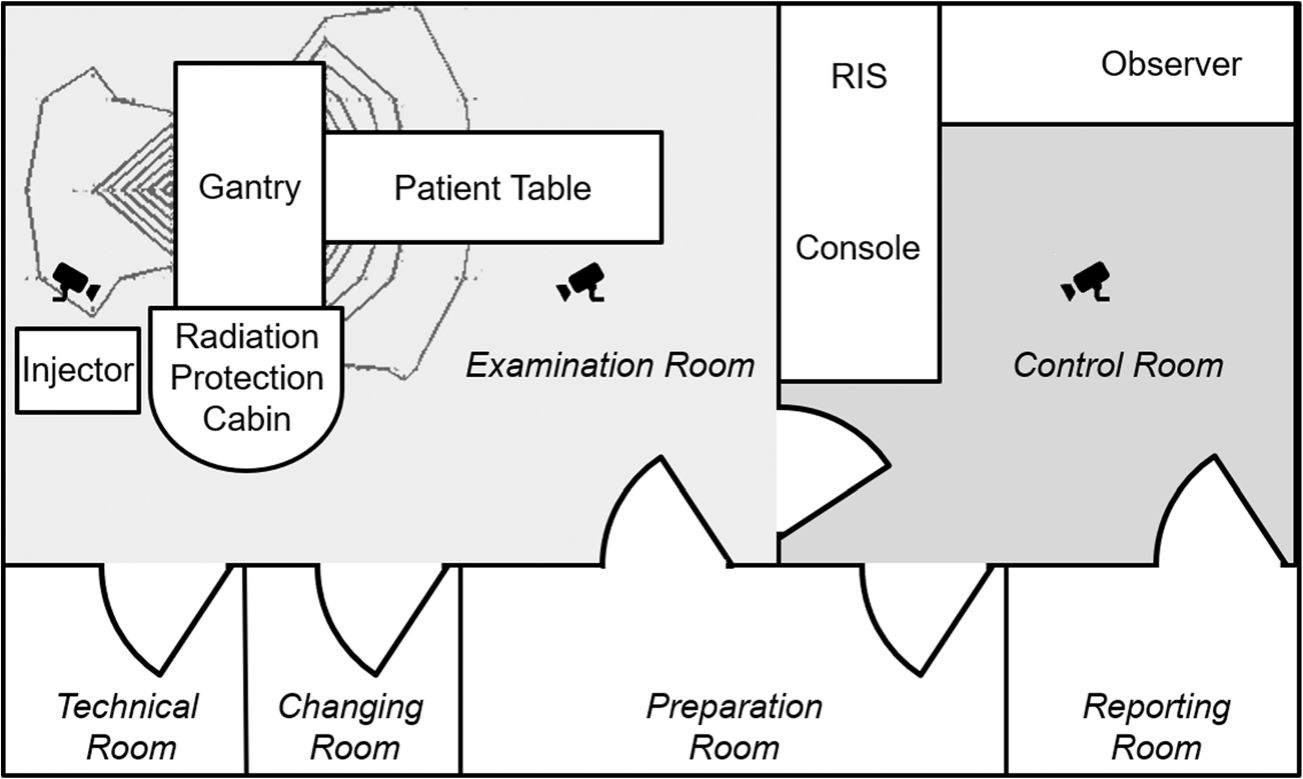
Schematic overview of the study setting in the examination room and in the control room (radiological information system, RIS). Isodose lines are sketched shaded black around the gantry. Positions of the ceiling mounted fisheye cameras are represented by the icons

A new CT system (Somatom go.Up, Siemens Healthcare GmbH, Forchheim, Germany) was installed in our radiological department which can be run by a conventional stationary console or a mobile tablet (Elite ×2 1012 G1, Hewlett-Packard Inc., Palo Alto, US-CA). The tablet was equipped with a 12-in. full high definition display and a 64Bit operating system (Windows 10 Professional, Microsoft Corp., Redmont, US-WA). The recently released operating system (Somaris 10, Siemens Healthcare GmbH, Forchheim, Germany) comes along with a new user interface on the console. An additional mobile application on the tablet enables the communication between the mobile device and the scanner. Its functionality is limited to operational functions including registration, planning, examination, automated reconstruction and automated archiving. This simplified mobile user interface guides the radiographer through a 7 click examination. For this study all planned reconstruction volumes from the study and the control were reviewed by the radiographer on the console as a quality check before automatically archiving the resulting series to the PACS (Fig. 1). The software’s automatic anatomical range detection on the localizer (‘Scan & go’ feature) and automatic anatomical alignment of the reconstructions (‘Recon & go’ feature) assisted the radiographer in both, the mobile and the conventional workflow. Patient table positioning and radiation release can be operated from a stationary control panel adjacent to the console or via an additional separate wireless remote control in the examination room. This enables the operator to stay within the examination room during the entire duration of the procedure, but with the drawback of scattered radiation dose. Hence, a prototype of radiation protection cabin with 3 mm lead equivalent walls (MSR Röntgentechnische Systeme GmbH, Jünkerath, Germany) was designed for the examination room in order to support this new option to stay with the patient. It was built up on a semicircular base area in the irradiation-shadow of the gantry with the lowest isodose calculation (Fig. 2). Radiation exposure during the maximum tube output at 120 kV was measured by the local supervisory board (TÜV SÜD) to be at the same level like in the control room (0.5 μSv/h) and below 1 mSv/y.

### Patients

During a period of 6 months 360 CTs of the chest were examined with the new CT system. The study was limited to examinations of the chest in order to avoid a bias on the workflow evaluation by other influencing factors, such as different positioning techniques, multi-region or multi-phase protocols. Additionally, CT of the chest is a frequent clinical routine examination that promised to provide a large patient collective. 98 of the 360 patients met the inclusion criteria of regular patients´ mobility (including wheelchair users), informed consent of video surveillance and available monitoring staff. We excluded patients that were confined to bed (like intensive care unit patients) in order to avoid patients’ capabilities becoming a bias between the collectives. Unavailable monitoring staff was the reason for the vast majority of excluded patients. All 98 monitored patients were randomized to examination with the mobile workflow in the study group (*n* = 47) or the conventional workflow in the reference group (*n* = 51). The dedicated flowchart of the study design is shown in Fig. 1.

### Video surveillance

Three fisheye video cameras were mounted on the ceiling of the control room and the examination room as shown in Fig. 2. Camera 1 covered the control room, camera 2 the examination room in front of the gantry, and camera 3 the examination room behind the gantry and the radiation protection cabin. Video recordings were manually started at the beginning of each examination and manually stopped at the end by an additional observer, who was positioned in the control room. The starting point was signalized by the radiographer if the following conditions were met: next patient sits fully prepared in the waiting room - no other persons than the maximum of one leading radiographer, the patient and one supportive radiographer if needed are in the examination room or control room - no pending tasks on the CT system. Completion of all data transfers to the picture archiving and communication system (PACS) and documentation in the radiological information system (RIS) was defined as endpoint. Minimum recommended duration of the video file was 1 h according to the vendor. Therefore, a single cumulative video file of all patients was created for each group. The software is not able to distinguish between different persons.

### Heat-maps

**Fig. 3:**
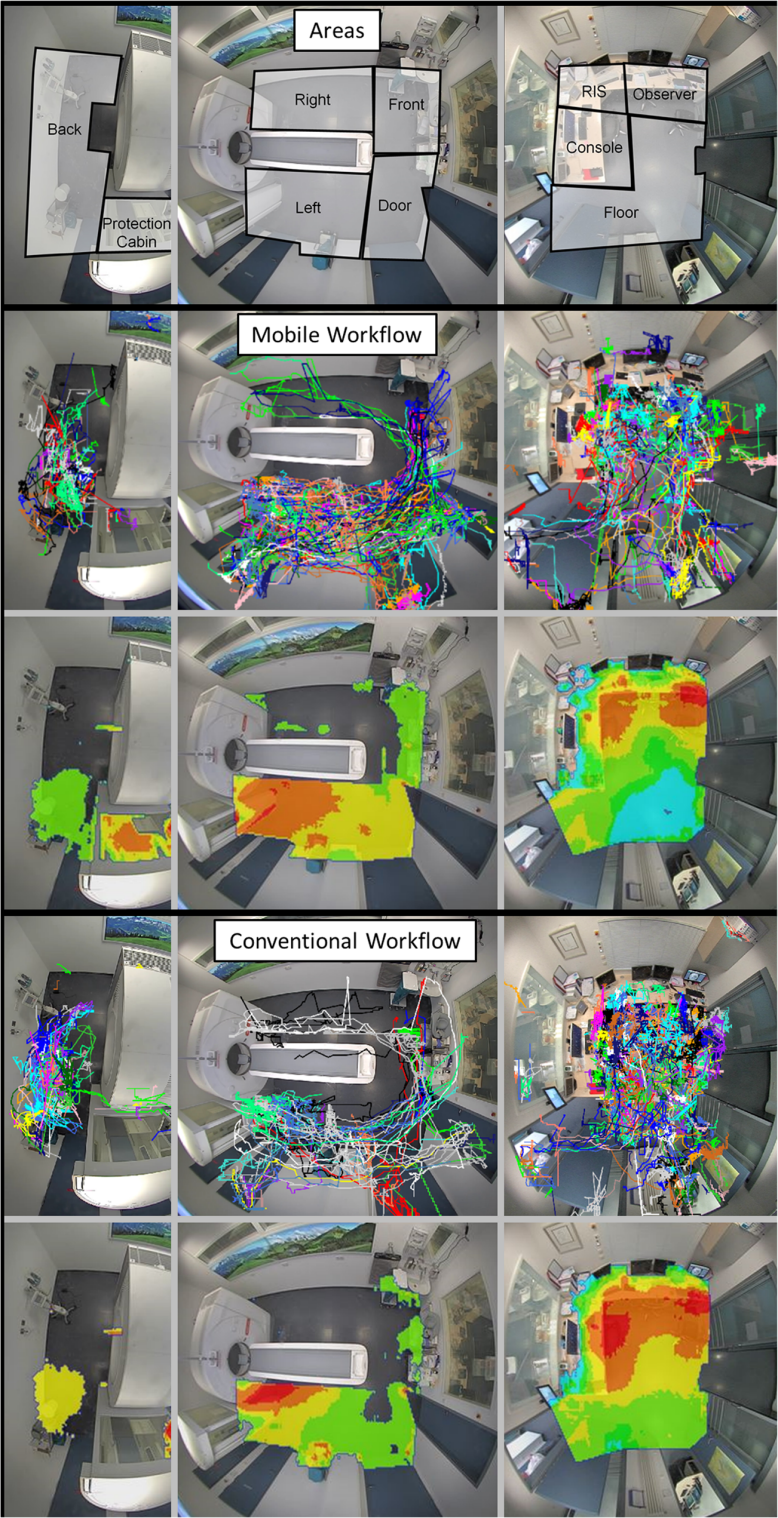
Perspective view of the fisheye cameras and sketched virtual areas for quantification of movement. Target lines (upper rows) are recorded and used as input for relative density function heat-maps (bottom rows) in false colours encoding higher (warm colours) or lower (cold colours) presence of persons in the examination room and in the control room

The video files were evaluated retrospectively using a dedicated software package for person recognition and motion tracking (VTrack, TechnoAware, Genova, Italy). Foreground videos, which are the basis for automatic video surveillance, were created by subtracting a background image from the video files [10]. Detected motion of a person was automatically tracked and target lines, representing trails of the moving subjects, were recorded (Fig. 3). Visualization of these traces over a timeframe can be done using relative density function heat-maps in false colors encoding the presence of persons during the video recordings for subjective evaluation. Warm colors in the heat-maps indicate high relative target line density (hot areas, red) and cold colors indicate low relative target line density (cold areas, blue) [11]. Areas that are clearly attributed to patients, like the patient table, and areas with light reflections, like mirrors and metallic surfaces, were excluded from the evaluation.

### Area-counts

Virtual areas within the cameras’ imaging areas were defined for quantitative evaluation as shown in Fig. 3. The control room was divided into four areas: floor (free area), console (interaction with the CT system), RIS (radiological information system for administration) and observer (coordinating the video recordings and time measurements). The examination room was divided into six areas: right and left (when viewed from the front of the gantry), front (of the table including the washbasin), door (free area between the patient table and the doors of the control room and preparation room), radiation protection cabin (monitored through its windows) and back (behind the gantry). Moving objects in those areas were automatically registered whenever a threshold was reached. This threshold was predefined by the vendor and could not be adjusted for this study. Total counts per area were referenced to the number of patients in the respective group to provide the mean counts per patient (cpp) following eq. 1:

$$ \mathrm{cpp}=\mathrm{total}\ \mathrm{area}\ \mathrm{counts}/\mathrm{sample}\ \mathrm{size} $$

### Time measurements

To support the validity of the video recordings, duration of all workflow tasks was simultaneously assessed manually by the observer using a simple stopwatch: registration, positioning, planning, contrast media injection, release, and post-processing. The examinations were mainly carried out and coordinated by one leading radiographer. When clinically needed, he was supported by a second radiographer in the mobile and the conventional workflow. Tasks fulfilled by the supporting radiographer were limited to patient transfer and management of contrast injections. However, extensive work-sharing was intentionally avoided. As soon as two radiographers were working simultaneously, a second stopwatch was started, and time measurements were noted separately and summed in order to avoid a bias to the complete duration of the examination.

### Statistics

All statistical analyses were performed using the software package SPSS Statistics Version 21 (IBM). Normality of distribution was tested using the Kolmogorov-Smirnov test. Median and range are provided in case of negative test results. Differences in time measurements between the study and the reference group, and subgroup analyses were carried out using the non-parametric Mann-Whitney-U test. The significance level was defined as *p* < 0.05.

## Results

A total of 527 and 677 min were recorded by each camera for the mobile and conventional workflow. Mean age of the patients was 60.2 years in the study group and 60.3 years in the reference. In the study group 64% of patients were male compared to 60% in the reference. Indications for CT of the chest in the study/reference group were malignant disease in 15/19 cases, infectious disease in 16/13 cases, chronic interstitial lung disease in 10/10 cases and others in 6/9 cases. 3 wheelchair users were included in the mobile workflow compared to 9 in the conventional workflow. Detailed patient characteristics and radiation dose are listed in Table 1. A second radiographer was needed for assistance in 14 examinations of the study group and 10 examinations of the reference. Median age of the 10 different radiographers (2 male/ 8 female), who performed the examination, was 29 years (range 20–62 years) and the median clinical experience in CT was 3.5 years (range 0.5–18 years).

**Table 1:** Patient characteristics

	Mobile	Conventional
Total number of patients	47	51
Male	30	31
Mean age (years)	60.2	60.3
Mean body mass index	25.5	25.1
Wheelchair user	3	9
Contrast media injection	10	24
Mean CTDI (mGy)	4.31	4.26
Mean DLP (mGy*cm)	171	156
Mean estimated ED (mSv)	2.4	2.2

### Heat-maps

Heat-maps of the radiographers’ movements provide a comprehensive overview of the radiographers’ location throughout the examination sequence (Fig. 3). Hot areas decreased in the control room, especially in front of the console and across the floor area. A remaining hot area is located in front of the RIS-system, for which a mobile integration is not yet available. The rather constant hot area in front of the surveillance monitor in the control room can be explained by the stationary interaction of the observer. In the examination room, the focal hot spot beneath the top of the patient table and the front cover of the gantry in the reference group evolved to a large hot area spread out over the entire left side in the study group. Also, the area around the injector at the backside of the gantry increased in size and decreased in density, most probably because of an increased freedom of movement using the tablet and remote control. Exemplary benefits from this increased freedom of movement that were found in the videos were: registration and identification while walking into the examination room - selection of the procedure while discussing symptoms with the patient - instruction of rough positioning while moving the table into a first position - precise patient positioning while preparing the contrast injection or fetching cushions - obtaining a view from the end of the patient table aligned to the gantry for precise adjustment of the table to the isocenter. Cold areas were registered in front of the patient table and on the right side in both collectives, but these few detections were also slightly higher in these areas for the mobile workflow. A new area of high relative target density occurred in the radiation protection cabin in the study group.

### Area-counts

**Fig. 4:**
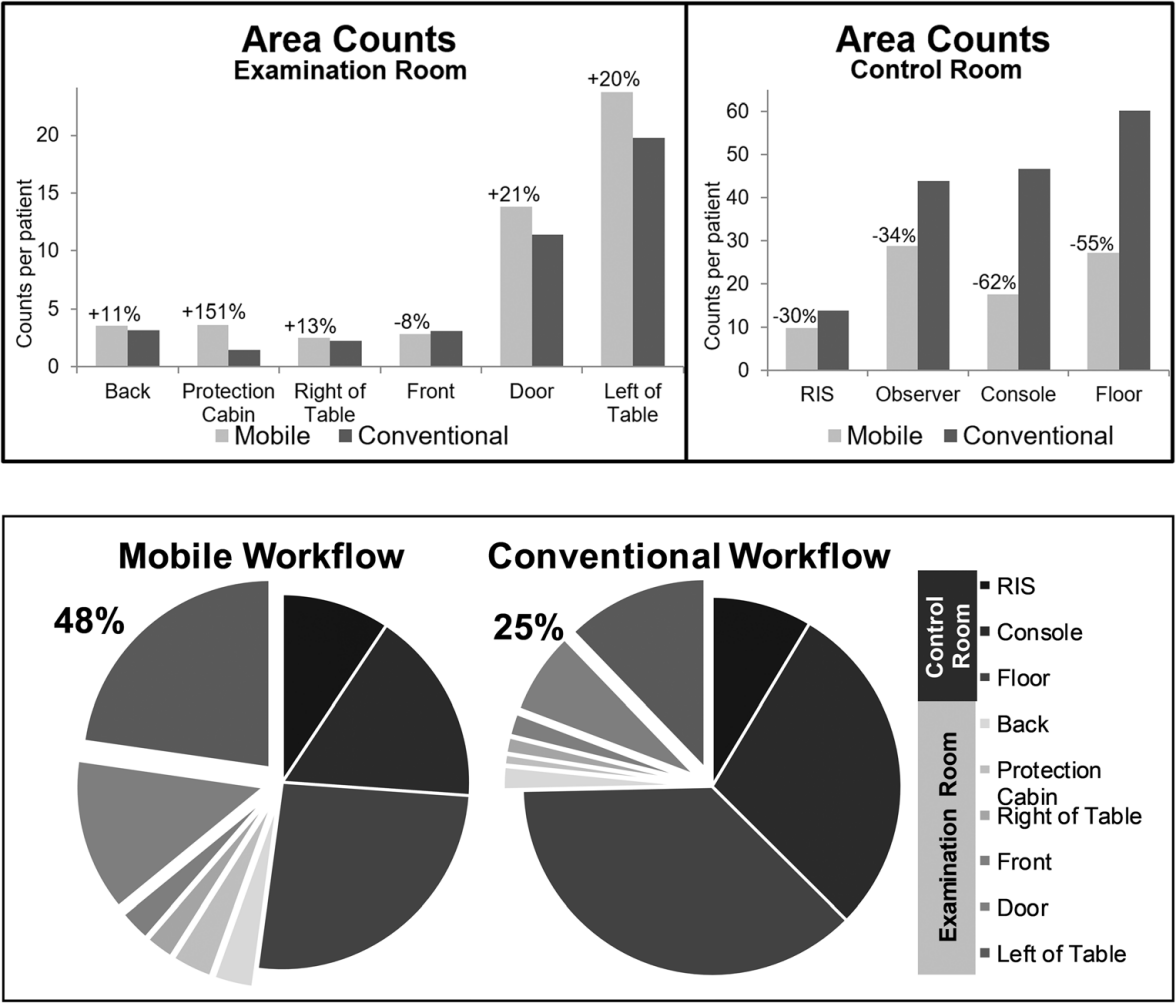
**a** Counts per patient in the predefined areas for the mobile and the conventional workflow in the examination room and in the control room. Relative changes are shown for the mobile workflow. Radiological information system (RIS). **b** Pie charts of area counts per patient of the mobile and the conventional workflow. Areas in the examination room are highlighted with an offset. The proportion of counts in the examination room relative to the entire examination is shown in numbers. Counts from the observer area were discarded in order to simulate a clinical routine setting. Radiological information system (RIS)

Area counts are shown in Fig. 4a. Highest number of counts per patient was found in the floor area of the control room for the reference group (*n* = 60 cpp) and in the observer area of the control room for the study group (*n* = 29 cpp). Lowest number of counts per patient were found in the radiation protection cabin for the reference group (*n* = 1 cpp) and on the right side of the table for the study group (*n* = 3 cpp). There was a substantial decrease of counts per patient in all areas of the control room for the mobile workflow. Highest reduction was observed in the console- (−62%) and in the floor-area (−55%). Reduction in the RIS-area was less (−30%). Overall area counts per patient in the examination room increased, especially in the radiation protection cabin (+151%), on the left side of the patient table (+20%) and in the door-area (+21%). Only little differences were registered in the low traffic areas at the end of the patient table (−8%), on the right side of the patient table (+13%), and on the backside of the gantry (+11%). Figure 4b provides an overview of area counts per patient in both rooms. Counts from the observer-area were discarded in order to simulate a clinical routine setting. The area counts per patient in the examination room, thus in the same room as the patient, are almost doubled using the mobile workflow (48% of all cpp) compared to the reference (25%). Counts per patient in the examination room excluding the radiation protection cabin, where staff is moderately separated from the patient by a leaden glass wall, accounts for 44% of all cpp using the mobile workflow compared to 24% in the conventional workflow. However, in this study more than one fourth of all counts per patient (26%) are still due to an interaction with stationary systems (console 17%, RIS 9%).

### Time measurements

**Fig. 5:**
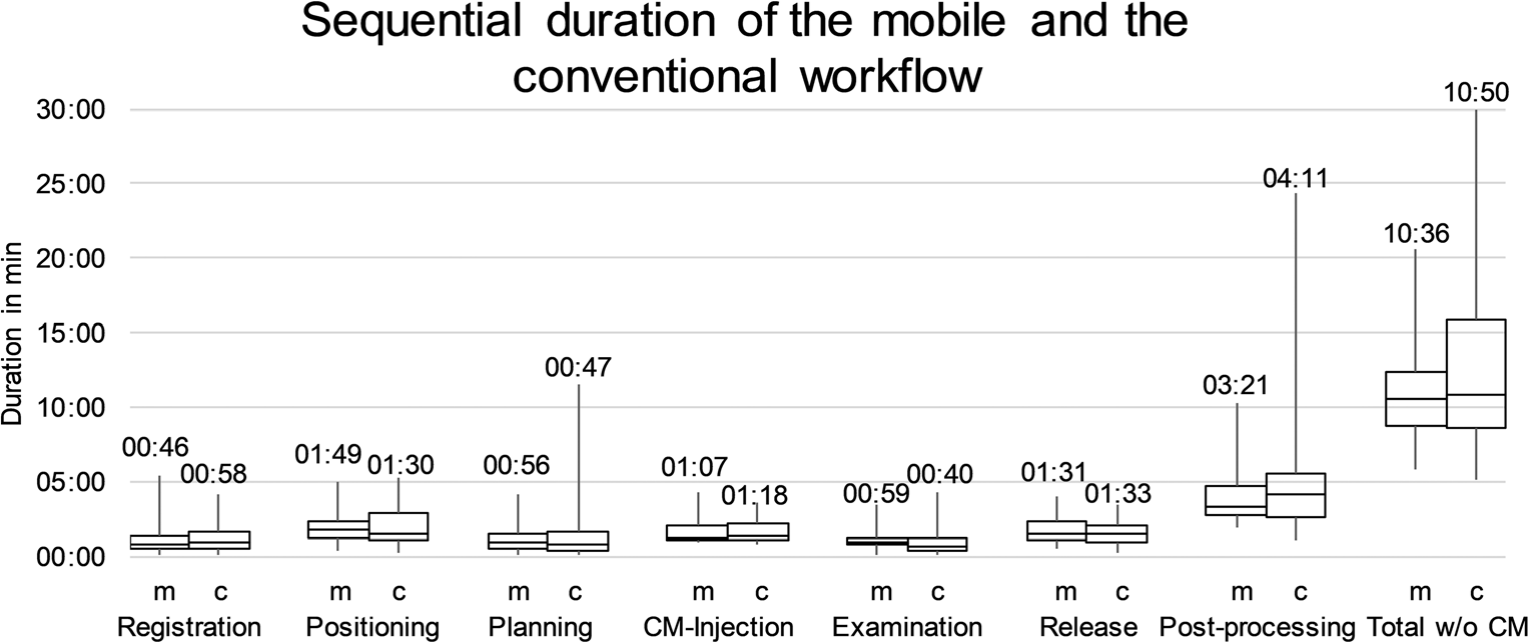
Boxplots of the duration of all parts of the workflow sequence for the mobile (m) and the conventional (c) workflow with the median duration per step displayed above. Contrast media (CM) injection was discarded for calculation of the total duration of the examinations

Median duration of the exams, excluding contrast media injection, was slightly lower in the study group (10:36 min, range 05:48–20:35 min) compared to the reference group (10:50 min, range 05:03–29:57 min) without statistical significance (*p* = 0.29, Fig. 5). Post-processing, that was done on the console in both collectives, was found to be the most time-consuming part of the sequence (study: median 03:21 min, range 01:52–10:17 min; reference: median 04:11 min, range 01:03–24:18 min) accounting for 31.6% and 38.6 of the total duration. However, differences were non-significant (*p* = 0.22). No statistical significance was found for the differences in all parts of the sequence as well (0.17 ≤ *p* ≤ 0.89). Median time spent in the same room with the patient increased from 3:06 min (28%) using a conventional setting (positioning and patient release sequences) to 6:01 min (57%) using the mobile devices (*p* < 0.05) because of the transfer of the registration, planning, and examination sequences to the examination room. Time spent in the radiation protection cabin was 00:59 min, representing 9% of the total duration. Therefore, the time together with the patient without any kind of separation (registration-positioning-planning-release, 05:02 min) accounted for 47% in the mobile workflow (p < 0.05). Duration of all parts of the sequence and the total duration of the examination were also comparable between the study group and the reference in the subgroup analysis of patients with and without contrast media injection (0.07 ≤ *p* ≤ 0.92).

## Discussion

Radiographers’ time spent in the same room with the patient, which we consider as a surrogate for patient contact and quality in patient care, increased if a CT of the chest was examined with the help of mobile devices. Contact to the patient could be easily increased without a mobile workflow if the radiographers simply take more time for the procedures in the examination room. The drawback of this approach would be an extended total duration of the procedure and therefore a reduced number of patients that could be done per day. The main advantage of the mobile workflow found in this study is that the time spent together with the patient in the same room can be almost doubled without extending the total duration of the examination. Usage of an additional radiation protection cabin adds another 9% of the total duration to patient-vicinity in voice and visual contact.

It is well known that the efficiency of a CT system is mainly limited to the clinical workflow [12]. Several studies evaluated different techniques like intelligent scheduling or multiple radiographer workflows to increase the patient throughput of a CT system in literature [13] [14]. However, to our knowledge, no approach has yet been presented to redesign the conventional workflow sequence. Lin et al. were able to show that physicians’ time spent with patients is a determinant of patient satisfaction [15]. Although we didn’t assess patient satisfaction, our experience from this study is that the increased proximity of the radiographers to the patients is also beneficial for the compliance during the examination, especially in critical cases like excited or confused patients. It is also well known that children’s compliance often depends on their parents to be in the examination room [16]. The in-room radiation protection cabin presented in this study could be used for these cases as well to minimize the time of separation and to stay in voice and visual contact without increased radiation dose burden. Moreover, parents could also shortly walk in and out the cabin between the localizer and the tomogram to further reduce the separation from their children.

The radiographers’ increased freedom of movement and in-room solution of a radiation protection area presented in this study also opens up for additional concepts to further improve the cost-effectiveness of CT, even if not yet proven by our results. Future software versions of the mobile application could increase in functionality, so that in its strongest form mobile devices could completely replace the stationary console. If also a RIS was integrated on a mobile device, completely new room concepts would be feasible. Architectural planning could then discard the entire control room in favor of a radiation protection area in the examination room. Interventional procedures could benefit from applications like the one for image guidance that has already been reported by Hirata et al. [17], or as interventional suites in general without requiring other additional equipment than the tablet screen and mobile remote control.

Some limitations must be respected when interpreting our result: First, the mobile workflow was completely new to our radiographers while the conventional workflow has already been well established. Hence, it remains unclear if the rather little reduction of the total duration is due to the increased patient interaction, to the software or room concept in general, or to adaptation problems with the new situation. We expect that a learning effect and further improvements of the mobile workflow, like for example implementing a post-processing option on the tablet, could lead to additional time savings. Second, radiographers that operate a CT cannot be blinded to the procedure. Third, the camera surveillance was unable to differentiate between different radiographers and patients. The second supporting radiographer and patients that had to cross over from the preparation or changing room to the patient table imply a measurement bias. We assume this bias to be small because the frequency of a second assisting radiographer was comparable (30% and 20%) and patients’ interference should be identical and very short in both groups. The erroneous detection during transfer to and from the patient table only takes a few seconds, which we consider negligible in relation to the total duration. Moreover, this bias should not be represented in the time measurements since a second stopwatch was started whenever the second radiographer participated in the examination. Fourth, active time measurements using a stopwatch introduce their own errors. However, we consider the clinical collective large enough to overcome these rather slight inaccuracies. Fifth, we included all consecutive chest CT examinations in order to obtain a high sample size, with the drawback that we are unable to provide information about a special disease or degree of mobility. Sixth, contrast-enhanced and non-enhanced studies cannot be separated in the video evaluation. The knowledge from the time samplings that the contrast application accounts for approximately 10% of the total duration should be respected for the interpretation of the subjective maps. Anyway, we assume that the asymmetric ratio in the study (native/contrast = 3.7) and the reference (1.2) group rather favors an underestimation of area counts in the examination room for the mobile workflow. Seventh, counts in the control room decreased in all areas for the mobile workflow, although stable counts in the ‘RIS Area’ and the ‘Observer Area’ were expected. We attribute this effect to an inaccuracy of the video surveillance software. Persons moving on the edge of one area might overlap into adjacent areas. Eighth, the video surveillance software is limited to a cumulative assessment. Hence, we are unable to provide other statistical evaluation than the presented descriptive values.

## Conclusion

Mobile workflows in CT of the chest transfers the radiographers’ interaction with the scanner from the control room into the examination room. There, radiographers’ freedom of movement and time spent in the same room with the patient is higher compared to the conventional setting, without affection of the total duration.
